# The influencing mechanism of big data analytics technology capability on enterprise's operational performance: The mediating role of data-tool fit

**DOI:** 10.3389/fpsyg.2022.948764

**Published:** 2022-09-23

**Authors:** Xiangmeng Huang, Shuai Yang, Junbin Wang, Fengli Lin, Yunfei Jiang

**Affiliations:** ^1^Department of Logistic Management, Business School, Changshu Institute of Technology, Changshu, China; ^2^Department of Management Science, School of Management, Fudan University, Shanghai, China; ^3^Department of Education, School of Educational Sciences, Jiangsu Normal University, Xuzhou, China

**Keywords:** BDA technology capability, enterprise operation performance, fit between data and tools, resource-based view theory, dynamic capabilities views

## Abstract

With the development of network technology, enterprises face the explosive growth of data every day. Therefore, to fully mine the value of massive data, big data analysis (BDA) technology has become the key to developing the core competitiveness of enterprises. However, few empirical studies have investigated the influencing mechanism of the BDA capability of an enterprise on its operational performance. To fill this gap, this study explores how BDA technology capability influences enterprise operation performance, based on dynamic capabilities theory and resource-based theory. It proposes the key variables, including the connectivity, compatibility, and modularization of big data analysis technical capability, enterprise's operational performance, and the fit between data and tools, to establish a model and study the correlation between the variables. The results highlight the mediating role of data-tool fit in the relationships between BDA capability and the enterprise's operational performance, which is a major finding that has not been underlined in the extant literature. This study provides valuable insight for operational managers to help them in mobilizing BDA capability for enterprises' operational management and improving operational performance.

## Introduction

Big data has not only become an important asset of enterprises but also a key business resource for enterprises. More enterprises begin to think about and explore how to use big data capabilities to improve their operational performance (Papadopoulos and Gunasekaran, 2018; Dubey et al., 2020). Big data is a collection of data in heterogeneous formats and is characterized by volume, variety, velocity, value, veracity, variabi1ity, and visualization (McAfee and Brynjolfsson, 2012; Abbasi et al., 2016; Seddon and Currie, 2017). It has become one of the important strategic resources for enterprises and has changed the way they compete. Enterprises have developed big data analytics (BDA) capability to acquire, store, integrate, analyze, and deploy the data and transform them into useful and valuable information so as to make correct decisions, increase productivity, generate knowledge, and upgrade innovations for improved operation performance (Acharya et al., 2018; de Vasconcelos and Rocha, 2019). In China, as early as 2015, at the fifth plenary session of the 18th CPC Central Committee, the “National Big Data Strategy” was put forward and the “Action Plan for Promoting the Development of Big Data” was released, which brought big data into the perspective of various industries and scholars and became the focus of social attention. For Chinese enterprises, the development of big data is the entry point and breakthrough of enterprise transformation, as well as an important way to improve the enterprise's operational performance, while the BDA capability of enterprises is the key to the application of big data. To improve the enterprise's operational performance, it is necessary to explore the role of the BDA capability of an enterprise on its operational performance.

Enterprise's operational performance has received significant attention from scholars for a long time (Rauch et al., 2009; Sirmon and Hitt, 2009; Boso et al., 2013; Engelen et al., 2015; Aydiner et al., 2019). More recently, some researchers argue that technological innovation is not only the key factor to achieve success in the global competitive market but also plays an important role in the enterprise's operational performance (Akter et al., 2016; G€olzer and Fritzsche, 2017; Wamba et al., 2017; Mikalef et al., 2020; Shahbaz et al., 2021b). Research exploring the influences of big data on the enterprise's operational performance is still in its infancy, especially the lack of empirical research. There is debate about whether and under what conditions such technologies can bring benefits to an enterprise's operational performance (Akter et al., 2016; Mikalef et al., 2019). It is argued that further analysis is required to bear out that investment in BDA could be a source of competitive performance for an enterprise, while there is some evidence showing BDA can create value (Sharma et al., 2014). Moreover, some researchers believe that more empirical studies are needed to explicate the mechanism of the diffusion effect of BDA on the improvement of an enterprise's operational performance (Marr, 2016; Günther et al., 2017). Bean surveyed Fortune 1,000 companies in 2017 and also showed that despite the enthusiasm for investing in big data, results varied widely in terms of success (Mikalef et al., 2020). These findings from research and practice show that for most companies, the mechanisms for realizing operational performance gains from BDA investments are unclear. Therefore, this study seeks to fill the above-mentioned research gaps by answering the following two key research questions:

(1) Does BDA capability have a positive impact on the operational performance of an enterprise? and
(2) Through what mechanism of mediating BDA capabilities is operational performance attained?

Furthermore, to advance this line of research, this study drew on resource-based view (RBV) theory and dynamic capability view (DCV) theory to investigate the mechanism of the influence of BDA technology capability on enterprise performance. Many scholars have paid attention to the conversion of resources into potential operational performance through the development of specific capabilities of an enterprise (Sirmon et al., 2011). The resource-based perspective attempts to explain how resources are transformed into capabilities and what actions are necessary to structure, bundle, and utilize the capabilities effectively while this resource-oriented view is seldom addressed (Barney et al., 2011). Besides, as the extension of RBV theory, DCV is believed to help explain how an enterprise maintains a competitive advantage in its operations in changing environments (Mikalef et al., 2018). Hence, this study can also bridge the research gap in terms of a more theoretical basis.

The literature has claimed that the diversity and breadth of data used by contemporary enterprises make the aspect of data quality significant, and IT strategists and data analysts are particularly concerned with the quality of the data they analyze (Chen and Zhang, 2014; Ren et al., 2017). Although data itself is important, enterprises need to possess the technology of the infrastructure capable of storing, sharing, and analyzing data. It is strongly believed that big data requires technologies that are capable of handling large amounts of diverse and fast-moving data, including innovative infrastructure and novel software and information system (Gupta and George, 2016). As a result, it is interesting and necessary to investigate whether the technology implied by an enterprise is congruent with its data source and further improves operational performance.

To answer the research questions above, we develop an integrated research model based on an in-depth analysis of BDA and highlight the impact of BDA technology capacity to investigate the enterprise's operational performance. Another aspect of this research is to examine the mediating role of data-too fit between the relationship between BDA technology capacity and an enterprise's operational performance. This signifies that the scope of this research is broader in predicting BDA and the enterprise's operational performance.

## Theoretical basis

### Big data analytics capability

It is argued that the meanings and concepts of big data (BA), BDA, and BDA capabilities (BDAC) change as these terms are used interchangeably (Mikalef et al., 2018). Some researchers defined BDA as the implementation of several analytical techniques that can process big data in all dimensions to produce actionable descriptive, informative, and illustrative results (Lamba and Dubey, 2015). More succinctly, Wamba et al. (2017) claimed that BDA refers to a systematic approach to analyzing and processing big data to create value. Due to the attributes of big data (as embodied by the 7Vs), it is also believed that traditional analytic tools or techniques may be ineffective for such data analysis so enterprises are compelled to make a considerable effort to extract significant insights from big data (Akter et al., 2016; Lai et al., 2018; Shahbaz et al., 2020). Thus, the notion of BDA capability has been proposed to broadly define the ability of an enterprise to leverage data management, technology, and talent to improve efficiency and effectiveness and increase competitiveness (Mikalef et al., 2018).

Early literature identified three main components of BDA capability, including organizational, physical, and human ability, among which organizational ability refers to BDA management strategies of analytics planning, sharing and coordination, investment, and control of analytics as a whole; physical ability refers to advanced IT infrastructure or technologies such as open-source platforms and cloud-based computing; and human ability is the analytics skill or knowledge of the data scientists who can understand the tools (Barton and Court, 2012; Davenport et al., 2012; McAfee and Brynjolfsson, 2012; Wixom et al., 2013; Kiron et al., 2014; Ransbotham et al., 2015; Wamba et al., 2015). Later, Akter et al. (2016) put forward three key building blocks of BDA capability as follows: management, technology, and talent capability. In a similar spirit, some scholars highlighted the three types of BDA capability: big data management capability to optimize models and decision-making; big data technology capability to deal with multiple data sources; and big data talent capability to perform assigned tasks in the big data environment (Wamba et al., 2017; Shamim et al., 2020).

### Resource-based view theory

Consumers' loss aversion is prompting consumers to evaluate expected benefits and expected risks before buying. According to Janis and Mann's (1977) earlier research, the “loss” caused by the hypothetical benefits and risks before the purchase behavior of consumers was referred to as expected regret, that is, the “loss perception” generated by the results that consumers expected to give up before the purchase decision was better than the results they chose. Kahneman et al. (1982) further divided expected regret into expected action regret and expected inaction regret. The former refers to the regret caused by consumers' expected purchase, while the latter refers to the regret felt by consumers when they give up the purchase. The further classification of anticipatory regret only measures the same concept from two different perspectives. To avoid the possible ambiguity caused by previous research descriptions, this study only explores the mechanism of impact on impulse purchases based on online consumer anticipatory inaction.

Marketing stimulus regulates the customer's psychological account and then induces customers to make online impulsive decisions. At the same time, loss aversion requires customers to take necessary self-control to reduce expected inaction and regret. There are three definitions of self-control: the first is the intuitive definition of the decision maker's ability to resist temptation; the second is the axiomatic definition of exerting preference relation on an alternative set; and the third is the definition of displaying preference (Jiang and Qu, 2016). Sharma et al. (2014) found that consumer impulse can be divided into three dimensions: cognitive flippancy, emotional indulgence and behavior lack of self-control, and self-indulgence and lack of self-control have no positive relationship with independent self-concept for consumers. Self-control can regulate impulsive behavior and decision-making, but self-control is a kind of limited resource. Once used, the resources that individuals rely on for other self-control will be reduced, which makes it difficult to reach the established performance standard of self-control and leads to the failure of regulating the individual's subsequent tasks (Dong and Ni, 2017). Studies have shown that self-control and long-term value orientation can help restrain consumption and increase willingness to thrift, but this effect is relatively obvious in the short term, while the long-term effect is not significant. Moreover, this effect is also regulated by the individual's material basis (Nepomuceno and Laroche, 2017). Self-control theory can explain why customers cannot resist the temptation of the marketing stimulus after they regret it and then make impulse purchases for Internet product again.

### Dynamic capabilities views

Though RBV has widely applied to highlight that the strategic advantage or operational performance could be enhanced of an enterprise when it has sufficient, valuable, rare, or imperfectly imitable resources and when the enterprise owns the capability to exploit the potential of resources; it failed to adequately explain why some enterprises attain a competitive advantage in situations of rapid and unpredictable change where resources and capabilities are subject to erosion (Eisenhardt and Martin, 2000; Cepeda and Vera, 2007; Felin and Hesterly, 2007). Thus, extending the RBV of the enterprise, the dynamic capabilities view (DCV) has been proposed, attempting to explain how an enterprise integrates, constructs, and reconfigures its resources and capabilities in volatile markets and changing environments (Teece et al., 1997). In addition, many researchers agree with the standpoint through their studies (Priem and Butler, 2001; Hitt et al., 2016; Gutierrez-Gutierrez et al., 2018).

Dynamic capability theory requires enterprises to analyze the constantly changing external environment by means of dynamic analysis so as to better allocate internal and external resources and capabilities of enterprises. This perspective stresses that resources alone without effective practical capabilities to relocate and integrate internal and external resources of enterprises are not sufficient to ensure a sustainable competitive advantage (Teece, 2007; Zheng et al., 2011). From this perspective, the competitive performance of an enterprise does not depend on dynamic capability itself but the allocation of resources by dynamic capability. Therefore, dynamic capabilities could be considered a strategic choice that allows an enterprise to update its existing operational capabilities as opportunities or needs arise (Pavlou and El Sawy, 2006; Mikalef et al., 2020). Furthermore, it is supported by theoretical and empirical studies, demonstrating that dynamic capabilities have a positive effect on enhancing operational performance by facilitating changes, specifically on the enhancement of technological capabilities (Zahra et al., 2006; Protogerou et al., 2012; Wilden and Gudergan, 2015). From the lens of DCV, enterprises that possess sufficient resources should also own the capabilities to determine the best time and ways to align and realign their internal and external resources with their strategy, without which BDA cannot be carried out effectively (Teece, 2014; Shamim et al., 2020).

### BDA and enterprise's operational performance

The relationship between BDA and operational performance has received significant attention from researchers. But as discussed above, whether and how BDA can bring benefits to an enterprise's operational performance still lacks discussion. Besides, as most of the existing research is focused on literature studies, empirical research is required. Table 1 summarizes and highlights the current literature on BDA related to the improvement of the enterprise's operational performance.

**Table 1:** Summary of literature on BDA related to enterprise operational performance.

**Author(s)**	**Methodology**	**Findings**
Dutta and Bose (2015)	Case Study (1 case)	To achieve operational efficiency and obtained improved decision making as informed by market
Ortega (2015)	Questionnaire to 50 SME owners	To reduce costs relating to manufacturing processes and to tailor strategies to fit each targeted customer segment and product or service
Morabito (2015)	Literature review	To achieve operational efficiency by cutting costs and to provide safer and more effective online transactions and security of supply chain
Palanimalai and Paramasivam (2015)	Literature review	To have better cost savings, to obtain enhanced security and result in optimal performance
Bernard (2016)	Case study	To achieve operational efficiency
Bravo and Appelkvist (2018)	Literature review, Interviews with 11 employees of 5 firms	To improve decision making capability
Das (2018)	Case studies, 5 Cases; Interpretive research paradigm	To increase operational efficiency and cost reductions, to improve business processes and to enhence co-creation
Elia et al. (2020)	Systematic Literature Review involving 49 articles	To generate more revenue, increasre productivity and obtain cost efficiency
Jayashankar et al. (2020)	Semi-Structured Interview with 10 respondents	To take optimal oprerational decisions and gain competitive advantages

Moreover, based on the basic resource view, Ghasemaghaei (2019) studied the main features of big data: the influence of data volume, data speed, and data diversity on corporate performance is studied, and the mediating role of data value and data accuracy is also studied. The study finds that different data characteristics have different impacts on corporate performance, and big data can affect operational performance through intermediate variables. Enterprises should invest in different big data features based on their resources and business strategies. Gupta et al. (2021) analyzed the role of BDA in an enterprise's operational performance by adopting qualitative analysis based on the theoretical framework of knowledge, contributing to the literature on the role of BDA in improving an enterprise's operational performance. Besides, Suoniemi et al. (2020) attempted to explain why investment in big data resources can improve enterprise performance and concluded that investment in big data resources can improve enterprise performance by improving the market-oriented ability of enterprises. On this basis, Ghasemaghaei and Calic (2020) discussed the relationship between the characteristics of big data (i.e., quantity, type, and speed), enterprise innovation performance, and overall enterprise performance in 2020. The research shows that the characteristics of big data should be separated operationally and conceptually and different characteristics of big data have different impacts on innovation performance.

Many theoretical results undoubtedly confirm the positive impact of BDA on an enterprise's operational performance, but most of them remain at the theoretical level, lacking certain empirical analysis. So Maroufkhani et al. (2020) collected data by using a questionnaire survey from 171 small- and medium-sized manufacturing enterprises in Iran. They analyzed the driving factors and results of implementing BDA in small- and medium-sized enterprises and finally confirmed the great influence of BDA on improving an enterprise's operational performance. It helps managers take effective and appropriate measures to use BDA.

## Conceptual framework and hypotheses

### Conceptual framework

Big data analysis is described as new technologies and architectures that can economically extract value from large and diverse data sets by enabling high-speed capture, discovery, and analysis (Mikalef et al., 2018). Thus, as a vital recourse of a modern enterprise, the innovative technological capability for processing information to obtain a better understanding of the data and lead to wiser decision-making (Hofmann, 2017). Moreover, it is claimed that traditional technologies have been replaced by big data processing tools to build up databases and information management systems. It is because big data should deal with unstructured, semi-structured, and structured data with more attention given to unstructured data as it constitutes 95% of big data (Ghasemaghaei, 2019). Thus, the enormity and complexity of big data from various sources in the changing environment call for a significant investigation of the BDA technology capability and to understand how it can help reveal useful insights from big data for better decision-making of an enterprise. According to Akter et al. (2016), BDA technology capability can be underpinned by three characteristics, including connectivity, compatibility, and modularity, which can reflect the level of enterprises' application of BDA technology capability. Therefore, based on RBV and DCV, this study constructed a conceptual model that allows the explanation of the enterprise's operational performance through information processing mechanisms enabled by using BDA technology capability and its three aspects.

**Figure 1:**
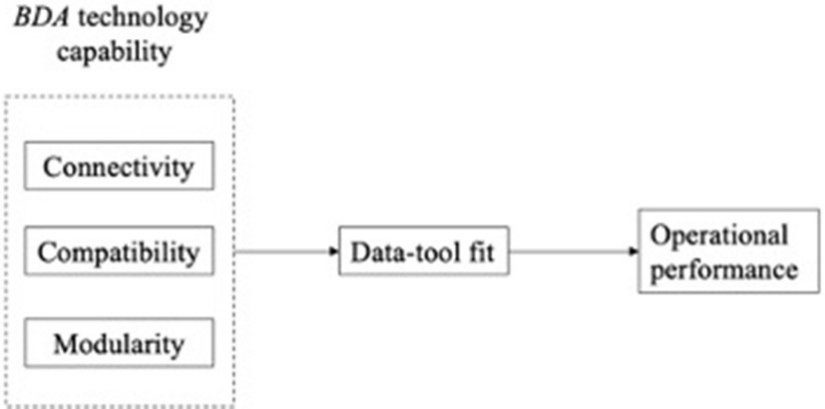
Conceptual framework.

In our conceptual framework, the links between BDA technology capability mastered by enterprises and their operational performances. It is outlined that BDA technology capability helps enterprises to deal with big data to make strategic decisions to improve operational performance. However, it is believed that BDA is influenced by the amount of data as well as the speed of data while the degree of data-tools fit affects the amount of data and the speed of data processing. Thus, the hypotheses are developed on the impacts of a mediating variable of data-tool fit. This study anticipated a mediating effect of the data-tool fit on the relationship between BDA technology capability and the enterprise's operational performance. Figure 1 presents the conceptual model as well as the set of hypotheses resulting from the discussion above.

### Hypotheses

The connectivity, compatibility, and modularity of BDA technology capability are of great importance to dealing with volatile business environments and allocating and integrating resources with enterprises' business strategies in the long and short term (Akter et al., 2016). Connectivity enables the effective sharing of information among different systems and applications. It empowers enterprises to source and connects various data points from remote, branch, and mobile offices for the improvement of their operational performance. For example, enterprises in the big data environment could improve customer service operations by combining data from the mobile terminal of their customers, online queries, social media comments, and customer complaints. In addition, compatibility refers to the ability to provide a continuous flow of information that can help create compatible data-sharing channels across various functions and for real-time decisions. It also facilitates cleanup operations that synchronize and merge overlapping data and repair missing information. For example, many enterprises have embraced compatibility in the BDAC platform by using cloud technologies or/and blockchain technologies that help in collaboration, experimentation, and rapid analysis. Furthermore, modularity refers to the ability to develop different functional modules, such as the application platform of big data analysis technology. These modules enable enterprises to delete, modify, and add systems and data as needed at any time when building data models, so as to help enterprises discover business opportunities more quickly. It also helps to develop business opportunities and improve the enterprise's operational performance. Hence, we proposed the following hypotheses:

H_1_: The connectivity of BDA technology capability has a significant positive impact on the enterprise's operational performance.

H_2_: The compatibility of BDA technology capability has a significant positive impact on the enterprise's operational performance.

H_3_: The modularity of BDA technology capability has a significant positive impact on the enterprise's operational performance.

Many scholars have stressed the importance of person-environment fit, which claims that enterprises' operational performances can only be improved when individuals are congruent with their work environment (Vogel and Feldman, 2009). Shahbaz et al. (2021a) also highlighted the fitness of technology with the task requirements of the users to obtain the successful adoption of BDA. While in the big data environment, when data, information, and technology play a significant role in implementing tasks and achieving good business performance, it is necessary to explore whether the technological tools are capable of handling certain data with high volume, variety, veracity, and velocity to extract valuable and authentic information and to generate efficient operational performance; that is, whether there is a data-tool fit that can contribute to the improvement of enterprises' operational performance. Based on the discussion above, the following hypothesis was proposed:

H_4_: Data-tool fit has a significant positive impact on an enterprise's operational performance.

In the big data era, the data, including texts, statistics, messages, weblogs, GPS location information, sensor data, graphs, videos, audio, and other online data, is becoming increasingly sophisticated, diverse, and changeable at any time. Thus, data requires different technological tools to collect, handle, and store (AlNuaimi et al., 2021). To obtain valuable data, for example, technological tools such as smartphones, RFID, cloud computing, the Internet of things, and blockchain technology are more suitable to be applied than traditional ways in terms of better real-time operational decisions and improvement of operational performance (Zhang et al., 2017). Therefore, though the BDA technology capability of an enterprise enables the availability of the various sources and profitable data, this data asset requires the deployment of “fit” tools capable of quickly analyzing those diverse big data in a scalable and precise way, which could support enterprises in improving their operational performance by increasing their profit and saving cost. Thus, we posited that the data tool will serve as a mediator of the relationship between BDA technology capability and the enterprise's operational performance.

H_5_: Data-tool fit has a mediating impact on the relationship between BDA capability and the enterprise's operational performance.

## Methodology

Our study aimed to investigate the mechanisms through which the technology capability of BDA impacts and contributes to an enterprise's operational performance in a complex context. To investigate the links and complementarities among the variables, we built a conceptual model and tested it empirically using a survey from 316 enterprises.

### Questionnaire design and variables

Based on the conceptual model, the survey was designed to cover each of the dimensions examined. The items of the questionnaire for the survey were adapted from previous research, and several studies were applied to operationalize the variables of the conceptual model. To measure the degree of agreement or disagreement with the questions asked, the five-dimensional Likert scales were used, which ranged from “1 strongly disagree” to “5 strongly agree.” All the variables used in the theoretical framework were applied as reflective items, which are presented in Table 2.

**Table 2:** Variables and measuring items.

**Variables**	**Measuring items**	**Statement**
Connectivity (Akter et al., 2016)	BDA1-1	Compared to our competitors in our industry, our enterprise has the most advanced analytical systems available
	BDA1-2	All remote office branches and mobile offices are connected to the central office for analysis
	BDA1-3	Our organization utilizes open systems network mechanisms to enhance analytical connectivity
	BDA1-4	There are no identifiable communication bottlenecks within our enterprise when it comes to sharing analytical insights
Compatibility (Akter et al., 2016)	BDA2-1	Software applications can be easily transported and used across multiple analysis platforms
	BDA2-2	Our user interface provides transparent access to all platforms and applications
	BDA2-3	Analytics-driven information is seamlessly shared across our enterprise, no matter where it is
	BDA2-4	Our enterprise provides multiple analytics interfaces or entry points to external end users
Modularity (Akter et al., 2016)	BDA3-1	Reusable software modules are widely used in the development of new analytical models
	BDA3-1	End users leverage object-oriented tools to create their own analysis applications
	BDA3-1	Use object-oriented techniques to reduce the development time of new analysis applications
	BDA3-1	The application can be adapted to meet the various requirements of the analysis task
Data-Tool fit (Vogel and Feldman, 2009)	D-T1	In our enterprise there is a good fit between the analytical tools we have access to and the data we process
	D-T2	The present analytical tools my enterprise has access to fulfill our data analysis needs
	D-T3	The analytical tools that my enterprise currently has access to provide pretty much everything that we need to analyze our data properly
Enterprise Operational Performance (EOP) (Srinivasan and Swink, 2018; Dubey et al., 2020)	EOP1	Delivery on time
	EOP2	Order fulfillment lead time
	EOP3	Inventory turnover ratio
	EOP4	Capacity utilization

### Sampling and data collection

A pilot test was carried out with five experts who had experience in the technology departments of enterprises. This allowed for clarification of the questions and items of the questionnaire survey. Moreover, three academics conducting research on the topic of BDA and operational performance were consulted to verify the variables and measurements. Their feedbacks were incorporated into the final survey, and the final survey respondents were assured that their identity would be kept confidential.

The questionnaire survey was used to test the hypotheses of our conceptual model. To build the measurement scale, a broad understanding of the theoretical basis and a large number of literature reading are needed. The research hypotheses and research models were proposed, and a questionnaire suitable for this study was designed. The initial questionnaire was prepared for the pilot test, the test results were analyzed, and the questionnaire was modified. Then questionnaires were distributed and collected online on a large scale, and the results were analyzed.

The survey was administered to respondents who had the responsibility for using BDA technology in their enterprises. The questionnaire survey was first designed to distribute questionnaires both online and offline, but due to the impact of the epidemic, only the online questionnaire for data collection was adopted finally. To ensure the universality of data, the questionnaire was distributed randomly in a large range within those enterprises that have already applied BDA. Questionnaires with leakage fill, fill in regularly, or inconsistent options were considered invalid. In addition, the objects of this survey were managers and employees of enterprises applying BDA technology. To maximize the quality of the questionnaire results, each respondent was preselected through closed questions concerning their knowledge of BDA in relation to their enterprises' operational performance. Invalid questionnaires should also be processed if there were obvious nonsuch staff members.

Of the 343 questionnaires distributed, 316 were completed and received from 316 individuals who are using BDA technology/tools in their enterprises. Thus, the final sample size was 316, which represents a response rate of 92.1%. In addition, the demographic profile of the respondents is shown in Table 3.

**Table 3:** Profile of research respondents.

**Factors**	**Categories**	**Sample (*N* = 316)**	**Percentage (%)**
Industry	Automobile	23	7%
	Energy	24	8%
	Biochemical engineering	18	6%
	Electronic & electric	27	9%
	Food	26	8%
	Steel	13	4%
	Petrifaction	8	3%
	Pharmaceuticals	22	6%
	IT	24	8%
	Real estate	13	4%
	Machine manufacturing	25	8%
	Service	42	13%
	Public institutions	51	16%
Enterprise size (number of employees)	1–99	105	33%
	100–499	98	31%
	500–999	65	21%
	1,000–2,999	22	7%
	3,000–7,999	11	4%
	>8,000	15	5%
Age of enterprise	< 1 year	30	10%
	1–3 years	79	25%
	3–5 years	63	20%
	5–10 years	74	23%
	>10 years	70	22%
Respondents' position	Senior management	17	5%
	Middle-level manager	62	20%
	Front-line manager	58	18%
	Executive staff	179	57%

## Data analysis

The testing of the research hypotheses in the conceptual model was based on hierarchical multiple regression. As it is more suitable for exploratory research, it is relevant for this study.

### Reliability and validity analysis

Table 4 shows the model fit coefficients, indicating the validity of the model construct and fit effect. It can be seen from the table that the value of the overall fitting coefficients, *X*^2^/*df* , is 2.0002, < 3, indicating that the model construction and fit effect are good. RMSEA is 0.056, < 0.08, indicating a good fit too. Besides, the values of GEI, NFI, CFI, IFI, and TLI were 0.914, 0.933, 0.965, 0.966, and 0.958, respectively, which were all >0.9, accounting for good results. Therefore, the connectivity, compatibility, and modularity of BDA capabilities fit well with the model of the enterprise's operational performance.

**Table 4:** Construct validity.

***X*^2^/*df***	**RMSEA**	**GFI**	**NFI**	**CFI**	**IFI**	**TLI**
2.0002	0.056	0.914	0.933	0.965	0.966	0.958

The measurement model was evaluated based on the reliability of the internal consistency and the converging validity of measurements associated with the constructs and the discriminant validity. The reliability of internal consistency was verified by Cronbach's alpha and composite reliability. All of the values of the model were >0.8, indicating a good level of reliability of the internal consistency of the model (refer to Table 5). Besides, the converging validity of the measurements is verified by examining the loadings of the measurements with their respective variables, as displayed also in Table 5. As can be seen from the factor load table, the factor loads of enterprise performance, connectivity, compatibility, modularity, and data tool matching are all >0.6, indicating that the measurement topic corresponding to each variable is highly representative. The values of CR are 0.8743 0.8629, 0.8706, 0.859, and 0.8378 (all of them >0.8), while the values of AVE are 0.6351, 0.6127, 0.6279, 0.604, and 0.6328, respectively (all of them >0.5); both of which are indicating a good convergent validity of the model.

**Table 5:** Reliability and validity.

**Variables**	**Measuring items**	**Factor loading**	**Cronbach's alpha**	**CR**	**AVE**
Connectivity	BDA1-1	0.861	0.854	0.8743	0.6351
	BDA1-2	0.860			
	BDA1-3	0.844			
	BDA1-4	0.771			
Compatibility	BDA2-1	0.871	0.854	0.8629	0.6127
	BDA2-2	0.826			
	BDA2-3	0.820			
	BDA2-4	0.818			
Modularity	BDA3-1	0.869	0.868	0.8706	0.6279
	BDA3-2	0.834			
	BDA3-3	0.855			
	BDA3-4	0.826			
Data-Tool fit	D-T1	0.878	0.843	0.859	0.604
	D-T2	0.871			
	D-T3	0.869			
Enterprise operational performance (EOP)	EOP1	0.831	0.882	0.8378	0.6328
	EOP2	0.867			
	EOP3	0.858			
	EOP4	0.880			

In addition, the discriminant validity was verified if three conditions are met. First, each relationship between latent variables should be significant; second, the correlation coefficient is <0.5; and third, the correlation coefficient is less than the AVE square root.

As shown in Table 6, the three conditions are all met, which indicates a good discriminant validity of our research model.

**Table 6:** Discriminant validity.

	**EOP**	**Connectivity**	**Compatibility**	**Modularity**	**Data-tool fit**
EOP	0.6351				
Connectivity	0.073***	0.6127			
Compatibility	0.077***	0.092***	0.6279		
Modularity	0.073***	0.084***	0.085***	0.604	
Data-Tool fit	0.074***	0.077***	0.077***	0.076***	0.6328***
The square root of the AVE	0.7969	0.7828	0.7924	0.7772	0.7955

* indicates *p* < 0.1, ** indicates *p* < 0.01, *** indicates *p* < 0.001.

### Model testing results

The significance of the structural relationships of the research model was examined by the method of hierarchical multiple regression, and the results are summarized in Table 7 to verify the hypotheses.

**Table 7:** Coefficients estimates.

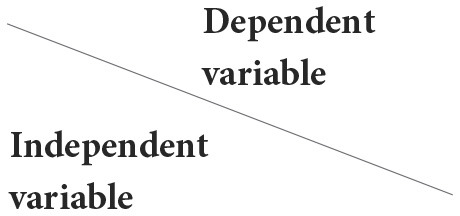	**Hypothesis 1**		**Hypothesis 2**		**Hypothesis 3**		**Hypothesis 4**	
	**β**	***t***	**β**	***t***	**β**	***t***	**β**	***t***
Connectivity	0.652	15.224***	0.194	2.877***	0.047	0.708	−0.045	−0.747
Compatibility			0.559	8.289**	0.383	5.593***	0.344	5.679***
Modularity					0.392	6.684***	0.187	3.331***
Data-tool fit							0.436	9.438***
*F*	231.772		175.231		148.011		164.618	
*R* ^2^	0.425		0.528		0.587		0.679	

^*^p < 0.1.

^**^p < 0.01.

^***^p < 0.001.

First, the impact of connectivity of BDA technology capabilities on an enterprise's operational performance is examined. *p* > 0.001, indicating that the significance level is high, and connectivity has a significant impact on enterprise operational performance. The determination coefficient *R*^2^ is 0.425, indicating that the goodness of fit is high and the model is well explained. The path coefficient β is 0.652, indicating that the connectivity of BDA technology capability has a significant positive effect on the enterprise's operational performance. So, hypothesis H1 is verified.

Second, we examined the impact of compatibility of BDA technology capabilities on an enterprise's operational performance. *p* > 0.01, indicating that the significance level is high, and compatibility have a significant impact on enterprise operational performance. The determination coefficient *R*^2^ is 0.528, indicating that the goodness of fit is high and the model is well explained. The path coefficient β is 0.559, indicating that the compatibility of BDA technology capability has a significant positive effect on the enterprise's operational performance. So hypothesis H2 is verified.

Similarly, the impact of modularity of BDA technology capabilities on an enterprise's operational performance is also significant. *p* > 0.001, indicating that the significance level is high, and modularity has a significant impact on enterprise operational performance. The determination coefficient *R*^2^ is 0.587, indicating that the goodness of fit is high and the model is well explained. The path coefficient β is 0.392, indicating that the modularity of BDA technology capability has a significant positive effect on the enterprise's operational performance. Thus, hypothesis H3 is verified.

The impact of data-tool fit on an enterprise's operational performance is then tested. *p* > 0.001, which indicates that data-tool fit has a highly significant impact on an enterprise's operational performance. The determination coefficient *R*^2^ is 0.652, indicating that the goodness of fit is high and the model is well explained. The path coefficient β is 0.436, indicating that the data-tool fit has a significant positive effect on the enterprise's operational performance. Hence, hypothesis H4 is also verified.

Finally, we used the process function of the SPSS software to test the mediating effect of data-tool fit between BDA technology capacity and enterprise operational performance, and the results are displayed in Tables 8, 9.

**Table 8:** Mediation model validation for data-tool fit.

	**EOP**		**EOP**		**Data-tool fit**	
	***t***	***p*-Value**	***t***	***p*-Value**	***t***	***p*-Value**
BDA	9.7498	0.0000	20.3989	0.0000	17.584	0.0000
Data-Tool fit	9.3983	0.0000				
*R* ^2^	0.6646	0.5699	0.4961
*F*	310.0853	416.1132	309.1971

**Table 9:** Proportion of mediating, direct and total effect.

	**Effect**	**BootSE**	**BootLLCI**	**BootULCI**	**Proportion**
Mediating effect of data-tool fit	0.107	0.017	0.077	0.143	40.47%
Direct effect of data-tool fit	0.158	0.021	0.115	0.198	59.53%
Total	0.265	0.014	0.238	0.291	100%

From Table 8, it can be seen that BDA technology capability has a significant positive effect on the enterprise's operational performance (*t* = 9.7498; *p* > 0.001). After the addition of data-tool fit as the mediator, the positive effect of BDA technology capability on an enterprise's operational performance is more significant (*t* = 20.3989; *p* > 0.001). Meanwhile, BDA technology capability has a significant positive effect on data-tool fit (*t* = 17.584, *p* > 0.001), and the positive effect of data-tool fit on an enterprise's operational performance is still significant (*t* = 9.3983; *p* > 0.001).

More specifically, Table 9 shows that the downline and upper limit of the confidence interval of the mediating effect of the data-tool fit were 0.077 and 0.143, respectively, excluding 0, indicating that the mediating effect was significant, and the proportion of the mediating effect and direct effect were 40.47 and 59.53%, respectively. Thus, data-tool fit plays a significant mediating effect between BDA technology capability and the enterprise's operational performance, and hypothesis H5 is verified.

Therefore, the relationships among the variables in the conceptual model were all verified to be significant, and the hypotheses test results are shown in Table 10.

**Table 10:** Hypotheses test results.

**No**.	**Hypotheses**	**Result**
H1	The connectivity of BDA technology capability has a significant positive impact on enterprise operational performance	Supported
H2	The compatibility of BDA technology capability has a significant positive impact on enterprise operational performance	Supported
H3	The modularity of BDA technology capability has a significant positive impact on enterprise operational performance	Supported
H4	Data-tool fit has a significant positive impact on enterprise operational performance	Supported
H5	Data-tool fit has a significant positive impact on enterprise operational performance	Supported

## Conclusion

### Discussion of findings

This study constructs a conceptual model with the enterprise's operational performance as the interdependent variable to explore the mechanism of how BDA technology capability can have an effect and the mediating effects of data-tool fit between the two variables.

First, BDA technology capability has a significant positive impact on an enterprise's operational performance through its features of connectivity, compatibility, and modularity. Based on RBV and DCV, BDA technology capability, as an important technological resource of enterprises, is also a dynamic and developable capability, and the flexibility of using BDAC can be changed through its three characteristics. Thus, enterprises should strive to learn and continuously develop this capability to establish and maintain their long-term core competitive advantages.

Second, data-tool fit plays a mediating role in the relationship between BDA technology capability and the enterprise's operational performance. The accuracy and rapidity of data are the keys to creating data value, and good tool matching will undoubtedly improve the flexibility of the application of BDAC, thus speeding up the speed of mining useful value from massive data, and thus improving the enterprise's operational performance.

This study also extends the theory of RBV and DCV by integrating BDA technology capability to achieve a deeper understanding of the data-tool fit of BDA technology in support of the enterprise's operational performance. Based on the underpinnings of BDA capability proposed by Akter et al. (2016), this study shows that an enterprise's operational performance can be improved with the connectivity, compatibility, and modularity of BDA technology. This finding reinforces the belief that enterprises with technological tools and data analytical capacity can mitigate the uncertainties and risks related to the interdependence among units and the dynamic environment of the market. It is an important contribution because the application of RBV and DCV in the study of an enterprise's operational performance, including delivery on time, order fulfillment lead time, inventory turnover ratio, and capacity utilization remains insufficiently investigated. Aligned with the perspective of RBV and DCV, the findings are also consistent with the discussion in the literature on the need for investment in technology innovation and big data resources to improve the operational performance of enterprises (Ghasemaghaei and Calic, 2020; Suoniemi et al., 2020).

### Theoretical contributions

There are two main theoretical contributions to this study. First, it demonstrates the impact of data-tool fit from the perspective of the internal system. In previous studies, some factors were considered as external moderating variables which could have an impact on the relationship between BDA-related variables and enterprise performance (Akter et al., 2016; Dubey et al., 2020; Horng et al., 2022). In our study, we proposed and verified that BDA technology capability needs to be internalized into data and the combination of appropriate tools to really play its role, and then promote the operational performance of enterprises. So our study contributes to the theory of the mechanism of the influence of BDA technology capability on enterprise performance. Second, many scholars usually measure BDA technology capability as one dimension when doing research and seldom analyze its connotation and characteristics. In this study, the BDA technology capability is divided into three dimensions for measurement, and the mechanism of the impact of these three dimensions on enterprise operational performance is analyzed and verified, respectively, which fills the theoretical gap in this aspect.

### Practical implications

This study also shows three implications. First, this study confirms the positive impact of BDA technology capability on enterprise performance, which is consistent with previous studies. Thus, enterprises must realize that in an era of highly developed Internet information technology, the digital economy is an inevitable trend. The application of BDA technology is crucial to the enterprise's operational performance and is the core of establishing long-term competitive advantages for enterprises. Therefore, enterprises need to adopt a positive attitude to introducing and applying BDA technology. Second, it is confirmed that BDA technology capability has a positive impact on an enterprise's operational performance, among which data-tool fit plays a mediating role. While no matter it is to master and develop BDA technology capability, or to complete the matching of data and tools, professionals are needed to carry out. Enterprises should introduce and train a large number of BDA technological personnel to form a corporate big data culture, to improve their BDAC in different directions. Besides, to improve data and tool fit, it is also suggested to adjust the organizational structure of the operational process of BDA technology application to strengthen the connectivity, compatibility, and modularity of BDA technology application and to improve the flexibility of BDA technology application in enterprises, so as to improve their enterprise's operational performance. Third, though BDA technology development is in full swing, there are still many enterprises that dare not invest in BDA technology. There have been many related literatures proving that BDA capacity has a positive effect on an enterprise's operational performance, and this study also confirmed the idea. In the increasingly competitive digital age, mastering BDA technology as soon as possible will help enterprises discover business opportunities faster and establish long-term competitive advantages. Therefore, enterprises must have the courage to invest in BDA technology and apply it to create value.

### Limitations of the study and future research

We believe that our model is sound, has a solid theoretical basis, and has been tested with reliable survey instruments and data. In addition to the contributions of this study, we recognize some limitations, and some future directions of research are proposed based on these limitations. First, the study was carried out within the specific domain of big data analytics and its impact on the context of an enterprise's operational performance was observed. Though BDA by its nature is context-specific due to the changing attribute of the data environment, replications of the conceptual model from future studies in other settings would enhance its generalizability. Second, the data collected for this research was from China, and the results of this research might change in a cross-cultural context. Future research could pay more attention to a multicultural context to increase the universality of the model. Third, the samples in this study came from different industries, and it could not be denied that the BDA capability and enterprise's operational performance in different industries have different characteristics, so the role of the BDA capability of enterprises in different industries is different. Therefore, it would be interesting for future research to further expand the ideas on other dimensions of BDA capability and enterprise-related indicators in the same industry. Fourth, we recommend investigating context-specific BDA dimensions (e.g., investment decision-making analytics, coordination, and control analytics) through a rigorous scale validation procedure to better measure BDA capability for various industries.

## Data availability statement

The original contributions presented in the study are included in the article/supplementary material, further inquiries can be directed to the corresponding author.

## Author contributions

SY contributes to supervision and the validation of this study. XH is responsible for writing—review and editing and formal analysis. JW contributes to the conceptualization, project administration, and validation. FL and YJ are responsible for methodology. All authors contributed to the article and approved the submitted version.

## Funding

This study is funded by the National Natural Science Foundation of China, No. 71901042, China Postdoctoral Science Foundation, No. 2021M690654, and the Major Project of Philosophy and Social Science Research in Colleges and Universities of Jiangsu Province, No. 2021SJZDA033.

## Conflict of interest

The authors declare that the research was conducted in the absence of any commercial or financial relationships that could be construed as a potential conflict of interest.

## Publisher's note

All claims expressed in this article are solely those of the authors and do not necessarily represent those of their affiliated organizations, or those of the publisher, the editors and the reviewers. Any product that may be evaluated in this article, or claim that may be made by its manufacturer, is not guaranteed or endorsed by the publisher.
